# Preparation and application of oyster shell supported zero valent nano scale iron for removal of natural organic matter from aqueous solutions

**DOI:** 10.1186/s40201-014-0146-y

**Published:** 2014-12-19

**Authors:** Vali Alipour, Simin Nasseri, Ramin Nabizadeh Nodehi, Amir Hossein Mahvi, Alimorad Rashidi

**Affiliations:** Department of Environmental Health Engineering, School of Public Health, Tehran University of Medical Sciences, Tehran, Iran; Center for Water Quality Research (CWQR), Institute for Environmental Research (IER), Tehran University of Medical Sciences, Tehran, Iran; Nanotechnology Research Center, Research Institute of Petroleum Industry (RIPI), Tehran, Iran

**Keywords:** Humic acid, Iron supported oyster shell, Nanoadsorbent, Natural organic matters, Adsorption, Isotherm

## Abstract

**Background:**

In this Research, oyster shell supported zero valent iron nanoparticles were prepared and applied for the removal of natural organic matters (NOMs) from aqueous solutions under different experimental conditions.

**Methods:**

The nanoadsorbents prepared by wet impregnation method, then characterized using Scanning Electron Microscopy, Energy Dispersive Spectroscopy, X-Ray Fluorescence and BET analysis. Adsorption test was done in a batch reactor and the effects of different parameters such as initial adsorbate concentration, adsorbent dose, adsorption kinetic, pH, and temperature on removal of NOMs (humic acid as the indicator) were studied.

**Results:**

Results showed that particle size of nanoadsorbent was in the range of 60-83 nm, and surface area and micropore volume as 16.85 m^2^/g and 0.021 m^3^/g, respectively; the main elements of adsorbent were Ca, O, Fe and Na and lime, as high as about 94.25% was the main structural component of the total weight. Produced nanoadsorbent was not soluble in water. It was also shown that by increasing the nanoadsorbent dose from 0.5 to 5 g/100 ml, the removal of humic acid increased from 62.3% to 97.4%. An inverse relationship was found between initial concentration and adsorption capacity, so that a decreasing rate of 33% for humic acid removal was observed by increasing pH from 5 to 10. Temperature increase from 25°C to 40°C, resulted in an increase in humic acid removal from 76.8% to 91.4% and its adsorption on the adsorbent could be better described by Freundlich isotherm (n = 0.016, K_f_ = 0.013 and R^2^ = 0.74). The most fitted adsorption kinetic model was pseudo-second order model.

**Conclusions:**

The chemical structure of nanoadsorbent was proper and free from harmful substances. Despite the relative good condition of the effective surface, due to the large size of the shell, the overall micropore volume was low. Hence the qualitative characteristics the adsorbent caused the absorption capacity of humic acid to be low (0.96 mg/g).

## Background

Although surface water is an important natural resource used for many purposes, especially drinking, this resource generally contain natural organic matters (NOMs) so many scientific references have pointed out that more than 1500 different organic compounds are suspected to be present in drinking waters [1,2]. Naturally organic materials, a nonhomogeneous mixture of complex organic compounds, including; humic acids (HA), lipids, proteins, hydrophilic acids, carboxylic acids, amino acids, polysaccharides, and hydrocarbons are invariably present in surface and ground water resources, in dissolved or colloidal forms [3]. Various environmental and health problems have been reported that may be related to the presence of NOMs in natural waters, including; (a) the potential of NOMs have to cause undesirable color and taste [4]; (b) NOMs contributing the reactions with heavy metals and biocides to yield high concentrations of these substances and enhance their transportation in water [5]; (c) NOMs role in increasing coagulant and disinfectant dose requirements that lead to more sludge amounts; (d) in treatment plants, NOMs react with chlorine to form harmful organic compounds [4]; (e) NOMs are important factors in fouling which affect various applications of membrane processes [6]; and (f) their tendency to compete with low-molecular weight synthetic organic chemicals and inorganic pollutants, reducing their adsorption rates and equilibrium capacities [7,8].

Considering harmful effects of NOMs on human health, these compounds should be eliminated from water before the chlorination process in drinking water treatment plants and it is important for the public health and drinking water industry to find reliable methods to remove a wide range of organic contaminants from water [9]. During the recent years, a large number of researches have been carried out on the NOMs removal from water in order to minimize its impacts on water quality [3,10-14]. Based on these researches, the most conventional options for NOM removal include membrane filtration and ion exchange [15,16], ozonation [17], biodegradation [18], ultrasound waves [19], adsorption, and coagulation [20]. Among the methods, membrane separation and adsorption were the most effective and available processes for removing NOMs from water [3]. High removal efficiency and no harmful by-products production are the main advantages for adsorption process and many kinds of adsorbents have been developed for the removal of humic acid from water.

During the past few years, nanoparticles have been proposed as a removal method for a wide range of pollutants from waters [21]. Some important nanoparticles in water treatment include metal-containing nanoparticles, carbonaceous nanomaterials, zeolites, and dendrimers [22]. As nanoscale zero-valent iron (NZVI) particles have unique reactive and sorption characteristics, they have received high attention for the treatment of contaminated waters [23,24].

Several studies have been conducted using various materials such as mesoporous silica beads [25], carbon and titanium dioxide [26], chitosan beads [27], ceramic membranes [12], kaolin [28], and betonite [13] as a bases for nanoparticles. In southern coasts of Iran, there is a considerable amount of oyster shells which may have the potential to be used for a perfect bases for nanoadsorbents, and so far no study and information are achieved on the use of the shell as adsorbent. Considering the possibility of successful production of air-stable ZVI nanoparticles with a high gravimetric ratio, good stability and no harmful matters, that in this research, it was studied as a bases for NZVI.

This study focused on the oyster shell supported NZVI application for the removal of NOMs under different experimental conditions, in order to remove taste and odor-causing agents from surface water resources.

## Methods

### Stock solutions

In this study, HA was used as NOMs representative and stock solution of HA was prepared using 1 g of HA powder dissolved in 1 L of deionized water, followed by filtering the solution through a 0.45 μ membrane filter (cellulose acetate). The solution was further diluted to the required concentrations and were stored at 4°C before application.

### Preparation of nano-supported shell

The formation of the oyster shell supported iron nanoadsorbent was carried out by wet impregnation technique [26-29]. Shells collected from the coast of the Persian Gulf were transported to research lab and after physically cleaning (removal of mud, rinsing for 10 min and washing by deionized water), were crushed, dried in 100°C oven and then sieved with a mesh of 100. To prepare the NZVI coated shell, the shell beads were placed in the reactor during the synthesis of NZVI, by using the reduction of dissolved iron method, which comprised of four stages; mixing, separating, washing and drying [30,31].

Initially, 5 g of crushed shell beads were poured in a flask containing 0.1 M FeCl3-6H2O dissolved in absolute ethanol and heated up to 80°C until the solvent was evaporated and dry coated beads were obtained. The beads were dispersed in 150 mL flask containing absolute ethanol and then the flask was placed on an orbital shaker. One hundred mL of 0.16 M NaBH4 aqueous solution was poured into a burette and dropped into stirring flask. During this reaction, ferric iron (Fe^3+^) was reduced to zero-valent iron (Fe^0^) by borohydride and the crushed shell beads started to take black color. This suggested that the ferrous ions attached to the support material were successfully reduced to zero-valent state, according to the following reaction:

1 $$ {\mathrm{Fe}}^{2+}+{{2\mathrm{B}\mathrm{H}}_4}^{-}+6{\mathrm{H}}_2\mathrm{O}\to {\mathrm{Fe}}^0+2\mathrm{B}{\left(\mathrm{O}\mathrm{H}\right)}_3+{{7\mathrm{H}}_2}^{\uparrow } $$

Afterwards, the contents of the flask were discharged to a funnel containing a millipore filter, under the suction force, leading to dewatering. The shell beads coated with NZVI were washed by ethanol, and then dried at 50°C and then kept in desiccators.

### Characterization of nanoadsorbent

Laboratory synthesized nanoadsorbent was analyzed using Scanning Electron Microscopy (SEM) of TESCAN, Energy Dispersive Spectroscopy (EDS) and X-Ray Fluorescence (XRF). The specific surface area of nanoadsorbent was analyzed using BET-N_2_ adsorption method.

### Batch reactor adsorption system and experiments

Adsorption of HA onto produced adsorbent was carried out by a batch reactor and effect of different parameters such as initial HA concentration, adsorbent dose, pH, and temperature were studied.

### Adsorption isotherm

Different doses of nanoadsorbent (0.5gr, 1gr, 3gr and 5gr) were added to the reaction flask containing 100 mL of 5 mg/L HA solution with the initial pH of 7 and temperature of 25°C. The containers were sampled: firstly at intervals of 5 and 10 min and then at a frequency of every 15 min to reach equilibrium time. After shaking and settling for 5 min, the samples supernatant were centrifuged followed by membrane filter (0.45 μm, cellulose acetate), absorbance values of solutions remaining without adsorption were measured by using UV–Vis spectrophotometer at wavelength of 254 nm. In the next step, adsorption equilibrium data were correlated with two well-known empirical isotherm models; Freundlich and Langmuir.

### The kinetic of HA adsorption

After determining the adsorption isotherm, using collected data and models related to the absorption kinetics, modeling kinetic of HA adsorption study on the nanoadsorbent was determined.

### The effect of initial HA concentration

The effect of initial HA concentration on the adsorption rate was studied by contacting 0.5 g of adsorbent at room temperature of 25°C and pH = 7 using four initial concentrations of HA solution (0.5, 2, 5 and 10 mg/L) in 100 mL of samples at time = 0 and at selected time intervals (up to a maximum of 180 min), sample concentration was determined by UV–Vis spectrophotometer.

### The effect of pH

In order to survey the pH effect on HA adsorption, pH of the solution was changed in the range of 5.0 and 10.0 at four intervals (5, 7, 8 and 10) using either 0.1 mol/L NaOH or 0.1 mol/L HCl. pH values were determined using a Elmetron CP-501 pH meter, fitted with a combined glass-reference electrode.

### The effect of temperature

In order to assess the effect of temperature on the equilibrium adsorption capacity of HA, 0.5gr of the nanoadsorbent was used at different temperatures (25, 30 and 40°C), with 5 mg/L HA solutions, optimum pH = 5 and in equilibrium contact time.

## Results and discussion

### Characterization of nanoadsorbent

**Figure 1:**
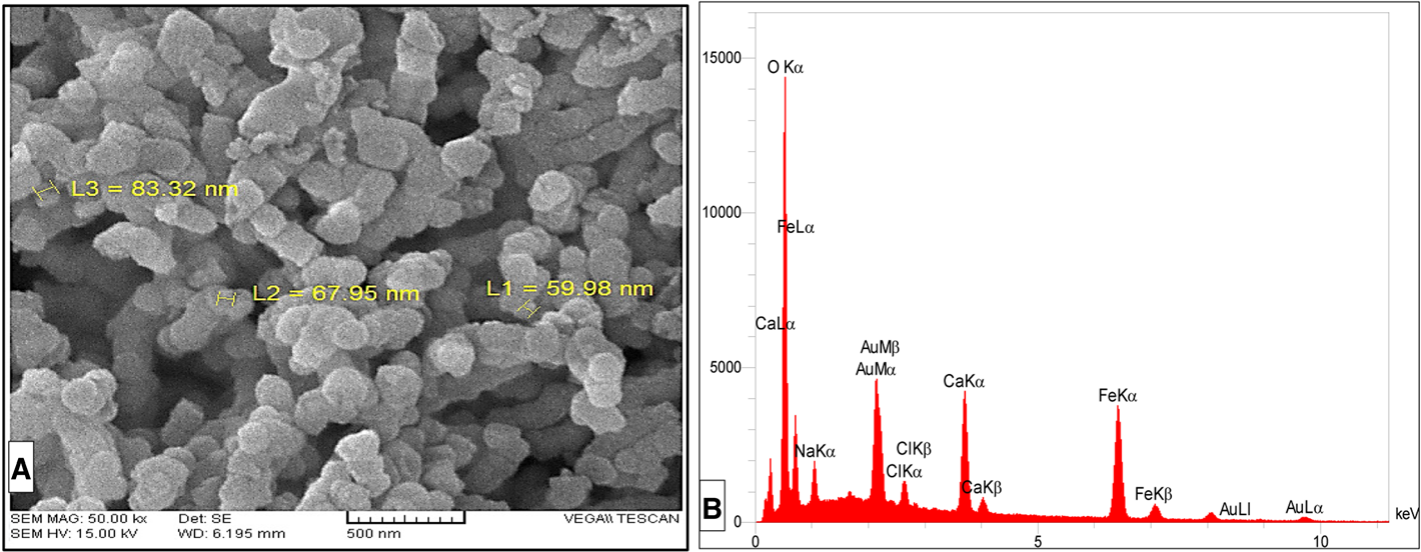
**SEM (A) and EDS (B) of prepared nanoadsorbent.** SEM **(A)** showing the Nanoscale particles and EDS **(B)** of prepared nanoadsorbent showing the main elements of adsorbent.

SEM image of the nanoadsorbent is shown in Figure 1, the physical properties of the nanoadsorbent are presented in Table 1, the chemical elements and composition of nanoadsorbent are presented in Tables 2 and 3, respectively.

**Table 1:** **Physical properties of the nanoadsorbent**

**Parameter**	**Unit**	**Value**
Density	g/cm^3^	1.82
Particle size	nm	60-83
Micropore volume	m^3^/g	0.012
BET surface area	(m^2^/g)	16.85
Solubility @ (20°C)	%	Insoluble

**Table 2:** **Chemical composition of the nanoadsorbent**

**Element**	**Weight %**	**Atomic %**	**Element**	**Weight %**	**Atomic %**
**O**	43.04	63.68	**Si**	4.1	2.23
**Na**	4.06	4.26	**Sr**	1.25	1.17
**Cl**	4.61	3.13	**Al**	2.3	1.64
**Ca**	17.1	10.28	**Mg**	0.78	0.43
**Fe**	9.46	4.08	**S**	1.5	1.95
**Au**	0.31	0.04	**K**	1.18	1.15
**C**	10.31	5.97	**Total**	100	100

**Table 3:** **Chemical composition of the nanoadsorbent**

**Composition**	**% Weight**	**Composition**	**% Weight**	**Composition**	**% Weight**	**Composition**	**% Weight**
SiO2	1.21	Al2O3	0.37	MgO	0.71	Na2O	1.01
CaO	21.09	K2O	0.65	Cl	0.07	SO3	0.19
FeOOH	4.01	CaCO3	63.38	SrO	0.81	Fe2O3	5.51

As shown in the Figure 1, most of the sheet structure has been changed to irregular small particles and depicts the synthesized nanoadsorbent with an approximately 60–85 nm diameter. The main elements of the adsorbent were Ca, O, Fe and Na and as it can be found from Table 3, the highest component of the adsorbents was lime (CaCO_3_ and CaO), whit about 94.25% of the total weight.

### The effect of the adsorbent dose

**Figure 2:**
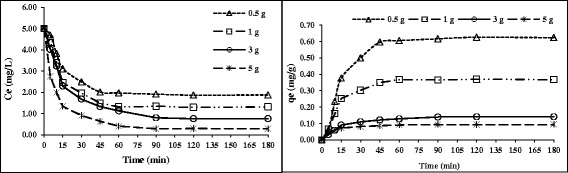
**The effect of the adsorbent dose @ (HA = 5 mg/L, Temp = 25** **°C and pH = 7).**

The effect of the initial HA concentration on its adsorption is shown in Figure 2. Although by increasing the nanoadsorbent dose of 0.5 to 10 mg/L, the removal of HA (C = 5 mg/L) was found to increase from 66.4% to 97.4%, the amount of removed HA (mg) per mass of nanoadsorbent showed a decrease in the rate from 0.66 mg/g to 0.09 mg/g. This is because of the fact that increasing adsorbent dosage increases the surface area for adsorption. However, very slow increase in the removal was observed beyond the dose of 3 g/L. This section of our results was in accordance with a study of Doulia etal who studied the adsorption of humic acid on acid-activated Greek bentonite [13].

**Figure 3:**
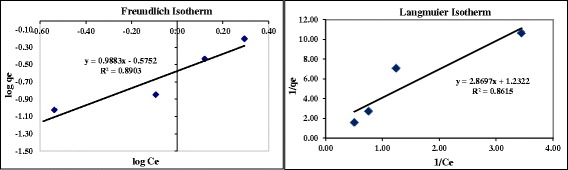
**Freundlich and Langmuir adsorption isotherms.**

The data of adsorption equilibrium for HA adsorption by nanoadsorbent are presented in Figures 2 and 3. As it can be seen, Freundlich isotherm had a better correlation with HA removal with R^2^ higher than Langmuir. The r-shaped adsorption isotherm indicated that there is a high affinity of contact time with adsorption rate.

### Kinetic of HA adsorption

**Figure 4:**
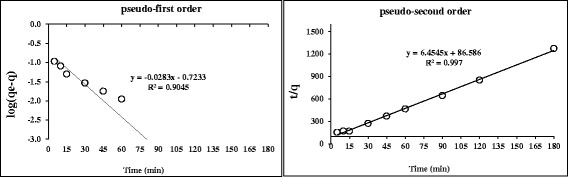
**Adsorption kinetics; pseudo-first order and pseudo-second order (nanoadsorbent dose = 3 g/100 mL, C0 = 5 mg/l, Temp = 25** **°** **C and pH = 7).**

Adsorption kinetics of HA as pseudo-first order and pseudo-second order are depicted in Figure 4.

Based on results of Figure 4, the pseudo second order model generates better fit to the experimental data of the investigated adsorption of HA on nanoadsorbent (R^2^ = 0.997). The degree of the reaction is determined by sharing or exchange of electrons between adsorbent positive (nzvi) and negative groups (HA).

### The effect of initial HA concentration

**Figure 5:**
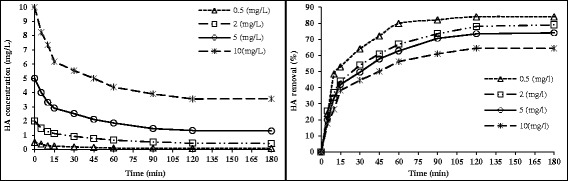
**The effect of initial HA concentration and contact time on HA removal by nanoadsorbent @ (nanoadsorbent dose: 0.5 g/100 mL, Temp = 25** **°C and pH = 7).**

The effect of initial HA concentration is depicted in Figure 5. It can be observed that the removal rate of HA decreased from 0.64 to 0.84, by increasing the initial HA concentration from 0.5 to 10 mg/L. The equilibration time for the adsorption of HA at different concentrations ranged between 90 and 120 min for 0.5 an10 mg/L of HA, respectively.

As can be seen from Figure 5 when an increase occurs in the initial concentrations, the adsorption capacity of HA would enhance. Driving force is one of the most important factors in the adsorption process so the adsorption capacity of HA is a function of HA initial concentration, as it is important to overcome the mass transfer resistance of the HA between the adsorbate and the adsorbent. In other words, the increase in the mass driving force allows more HA molecules to pass from the solution to the adsorbent surface. Therefore there is an inverse relationship between initial concentration and adsorption capacity [32]. This finding were in accordance with Moussavi et al [3] who investigated adsorption characteristics of HA onto single-walled carbon nanotubes and reported that the amount of adsorbed HA was higher at lower initial concentrations of HA.

### The effect of pH

**Figure 6:**
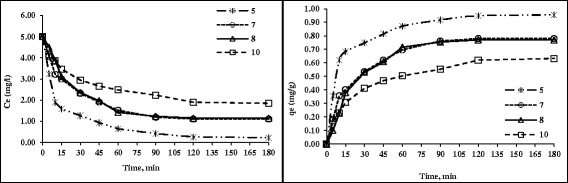
**The effect of pH and contact time on HA removal by nanoadsorbent (HA = 5 mg/L, nanoadsorbent dose = 0.5 g/100 mL and Temp = 25**°**C).**

The effect of the initial pH on adsorption rate is presented in Figure 6. As it can be seen, a maximum HA adsorption rate of 0.96 mg per gram of adsorbent was observed at pH = 5. This was followed by a decrease in adsorption capacity at the late stage of pH experiments ranging from 5 to 10. Also the minimum adsorption capacity was found to be 0.63 mg/g at initial pH = 10 after reaching the equilibrium time (120 min). Adsorption of HA to nanoadsorbent is strongly influenced by pH. As increasing the pH increases the ionization of HA and hence the concentration of the negatively charged ions which leads to decreasing the amount of H^+^ ions [33].

### The effect of temperature

**Figure 7:**
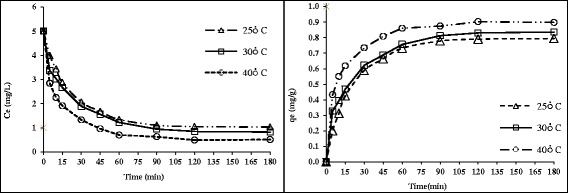
**The effect of temperature and contact time on HA removal by nanoadsorbent (HA = 5 mg/L, nanoadsorbent dose = 0.5 g/100 mL).**

The temperature effect on the HA adsorption on nanoadsorbent is shown in Figure 7, with HA concentration of 5 mg/L, the adsorption rate increased from76.8% to 91.4%, by increasing the temperature from 25°C to 40°C. As depicted in Figure 7, temperature increase led to further the HA adsorption, due to high interaction between HA and ZNVI-shell beads. The temperature increasing creates swelling effect in the internal structure of the adsorbent, causing further penetration HA into the adsorbent [34]. In addition, temperature increase causes more diffusion rate of HA molecules in the external boundary layer and the internal pores of the adsorbent particles. Our finding was in accordance with Zeinali etal results on adsorption of dichloromethane using GAC [35].

## Conclusions

The obtained results revealed that surface area and pore volume of nanoadsorbent were 16.85 (m^2^/g) and 0.021 m^3^/g, respectively; the main elements of the adsorbent were Ca, O, Fe and Na and most of the adsorbent component was composed of lime (CaCO_3_ and CaO about 94.25% of the total weight). The produced nanoadsorbent was not soluble in water. HA Adsorption onto the nanoadsorbent increased by increasing of adsorbent dose and temperature and decreased by increasing HA concentration and pH. The r-shaped adsorption isotherm indicated a high affinity of contact time with adsorption rate. In optimal conditions provided in the study (HA = 5 mg/L, nanoadsorbent dose = 0.5 g/100 mL, pH = 5 and temperature = 40°C), maximum HA removal efficiency reached to 94.6%, within 90-120 min at the experimental conditions in all cases is Pseudo-second order and Freundlich isotherm models were fitted to the adsorption kinetic and isotherm, respectively.
